# Medium-Term Lag-Response Associations Between PM_10_ Exposure and All-Cause Mortality in Valencia and London: A Time-Stratified Case-Crossover Study

**DOI:** 10.1007/s44197-025-00459-x

**Published:** 2025-08-30

**Authors:** Bin Zhou, Katrin Gohlsch, Surendra Ranpal, Jiancong Wang, Christoph Knote

**Affiliations:** Chair of Model-Based Environmental Exposure Science, Faculty of Medicine, University of Augsburg, Augsburg, Germany

**Keywords:** PM_10_ exposure, Medium term lag response associations, Lagging time windows of 21 days, All cause mortality, Environmental epidemiology, Temporal lag methodological structures, Europe

## Abstract

**Background:**

Air pollution is among the top five environmental risk factors for human health worldwide. However, our understanding of the physiological responses to PM_10_ exposure over medium-term lag periods remains limited. This study aims to examine the medium-term lag–response associations—using lagging time windows of up to 21 days—between PM_10_ exposure and all-cause mortality in Valencia and London from 2002 to 2006.

**Methods:**

We used a time-stratified case-crossover design; building on the methodologies of Tobias et al. and Bhaskaran et al., we applied a fixed-effects conditional quasi-Poisson regression model to quantify the association between PM_10_ exposure and all-cause mortality. We also analyzed three different temporal lag methodological models for the exposure–mortality relationships**.**

**Results:**

We found distinct differences in the relative risk (RR) patterns of PM_10_ exposure and all-cause mortality. In Valencia, the RR varied significantly, with confidence intervals that were wider than in London, where the RR remained more stable, fluctuating closely around 1. Significant associations were observed at early lag periods in both cities, consistent with Tobias et al. Notably, Valencia showed a significant peak in RR at lag 14, which was not observed in London. Subgroup analysis in Valencia also indicated delayed effects in younger populations. Scenario 3 (cumulative lag model) is conceptually closer to the cumulative progression of health risks associated with PM_10_ exposure and produces higher RR estimates compared to Scenario 1 and 2.

**Conclusions:**

This study highlights the critical importance of addressing medium-term lag-response associations and methodological variations in environmental epidemiology. The findings have important clinical and public health implications and offer insights for risk assessment, healthcare planning, and the development of policies to mitigate the health impacts of PM_10_ exposure.

**Supplementary Information:**

The online version contains supplementary material available at 10.1007/s44197-025-00459-x.

## Introduction

According to the European Environment Agency, air pollution is the leading environmental health risk in Europe, causing cardiovascular and respiratory diseases as well as preventable deaths [1, 2]. The level of PM_10_—mass of particulate matter with a diameter of 10 µm or less—is one of the most used metrics internationally for assessing outdoor air quality. According to the International Agency for Research on Cancer, PM_10_ is classified as carcinogenic to humans [3], although its carcinogenic potential also depends on its chemical composition. In 2021, the World Health Organization (WHO) published global air quality guidelines, recommending an annual average exposure limit for PM_10_ of 15 µg/m^3^ and a 24-h average exposure limit of 45 µg/m^3^ [4]. PM_10_ includes both PM_2.5_ (mass of particles with diameters of 2.5 µm or less) and PM_10–2.5_ (mass of particles with diameters between 2.5 and 10 µm), and its atmospheric lifetime ranges from hours for PM_10–2.5_ to several weeks for PM_2.5_ [5, 6]. From a medical perspective, acute exposure to elevated levels of PM_10_ can lead to increased hospital admissions for cardiovascular or cerebrovascular events, while chronic exposure increases the risk of non-communicable diseases and even, indirectly, death [7–9].

Environmental epidemiologists have long investigated the association between air pollution and health outcomes, not only to assess health impacts and progressive deterioration, but also to provide up-to-date research findings to inform health policies [9–11]. A recent systematic review from 2024 by Orellano et al. contributed to the updated WHO Global Air Quality Guidelines and was based on high-certainty evidence from 28 effect estimates [11]. The review reported a significant association between PM_10_ exposure and all-cause mortality, with a relative risk (RR) of 1.08 (95% confidence interval [CI]: 1.05–1.11). This indicates worsening health risks and an increase in the RR of all-cause mortality compared to a previous systematic review published in 2020 by Chen et al., which reported a pooled RR of 1.04 (95% CI: 1.03–1.06) [9, 11]. The increase in all-cause mortality risk between those two reviews could partly reflect an apparent increase in risk over time, although other factors such as differences in study populations, exposure assessment methods, and study periods may also contribute. This reinforces the need for public health interventions to mitigate air-pollution-related health impacts [5].

However, a gap remains in the research on PM_10_ exposure and all-cause mortality [9, 11], as most studies do not account for medium-term lag-response associations (e.g., lagging time windows of 21 days for risk estimations). Incorporating medium-term lag-response associations is clinically justified, as hospitalization and mortality due to air-pollution-related diseases often do not occur immediately after exposure. Instead of being solely driven by acute exposure, such adverse health events may peak 2–3 weeks later due to cumulative physiological damage, particularly for chronic diseases with exacerbations, recurrences, or decompensations [12]. While previous studies have primarily focused on short-term lag-response associations (lagging time windows of a week or less) [10, 13, 14], understanding medium-term lag-response associations is crucial for medical practitioners and public health stakeholders to help with hospital preparedness for patient admissions, early-warning systems and alerts, increased awareness campaigns for high-risk populations, and implementation of preventive measures to reduce PM_10_ exposure. Such efforts may collectively help delay or mitigate preventable adverse health outcomes [15, 16].

In addition to investigating the medium-term lag-response associations between PM_10_ exposure and mortality, it is methodologically important to compare computational models with varying lag structures [10, 14]. Simplified analyses may overlook subtle variations in lagged effects, potentially obscuring the true dynamics of the exposure–response relationships [10]. Bhaskaran et al. [14] introduced methodological concepts for modeling lag structures, including (i) treating lag effects as independent by fitting each lag into the independent model separately, and (ii) treating lag effects as joint effects by modeling all lag days together using an “unconstrained” distributed lag model. Tobias et al. [10] mainly focused on the latter approach, modeling all lag days together, but both studies primarily addressed short-term lag-response associations [10, 14].

To go beyond these approaches [10, 14], we proposed a methodological comparison of medium-term lag-response analysis using three different lag structures: (i) independent lag effects [14], (ii) joint lag effects [10, 14], and (iii) stepwise cumulative lag effects. In environmental research, there is currently no universally accepted “gold standard” for selecting the most appropriate lag structure for modelling. Our study therefore explored these three lag structures and their public health relevance to provide a comprehensive understanding of the temporal dynamics in the RR associations between PM_10_ exposure and all-cause mortality. This comparison aims to ensure that important nuances are not overlooked.

For this study, we used publicly available datasets from the metropolitan cities of Valencia, Spain and London, the UK. The aims were: (i) to examine the medium-term lag-response associations between PM_10_ exposure and all-cause mortality, and (ii) to investigate these effects using lagging time windows of 21 days across the three different lag methodologies.

## Materials and Methods

### Original Data Source

We used publicly available open-source datasets, which can be downloaded from Tobias et al. [10, 17]. These datasets are structured as date-level records, documenting the total number of all-cause mortalities in the respective cities on each specific date, along with 24-h average mean temperatures, relative humidity, and PM_10_ concentrations. The date-level data covers the period from January 1, 2002, to December 31, 2006. We treated the Valencia and London data as separate datasets for the analysis.

Daily all-cause mortality was simply the total deaths each day for 1,826 days. In the Valencia dataset only, mortality counts were divided into two age groups—less than 65 years and 65 years or older—for subsequent subgroup analysis [10].

Temperature, relative humidity, and PM_10_ data for Valencia were obtained from the National Institute of Meteorology and the Valencian Community Air Pollution Monitoring Network. For London, temperature and relative humidity data were sourced from the British Atmospheric Data Centre and PM_10_ concentration data were retrieved from the Westminster-Marylebone site of the London Air Quality Network [10].

### Study Design and Research Outcome

This study employed a time-stratified case-crossover design. The primary outcome was the RR associations between PM_10_ exposure and all-cause mortality in both Valencia and London, accounting for medium-term lag-response effects.

### Inclusion and Exclusion Criteria

We included all available data on daily all-cause mortality counts and environmental exposure from 2002 to 2006. No data were excluded.

### Statistical Analyses

We used the methodological concepts outlined in Tobias et al. and Bhaskaran et al. [10, 14], in our medium-term lag-response association analyses by applying a fixed-effects conditional quasi-Poisson regression model to quantify the associations between PM_10_ exposure and all-cause mortality in both Valencia and London. The *gnm* function from the *gnm* package in R was used to fit generalized nonlinear models. All the statistical analyses accounted for the RR association estimates up to 21-day lagging time window, with corresponding 95% CIs (Supplementary Material 1).

#### Case-Crossover Approach

We compared ambient PM_10_ exposure concentrations on days on which mortality occurred (termed *case days*) with exposure on days during the same month and on the same day of the week (termed *control days*) [10, 18, 19]. This approach was methodologically distinct from patient-level study designs.

#### Time-Stratified Approach

We constructed a categorical stratum variable for model fitting by interacting the year, month, and day of the week for each observation. This time-stratified approach accounted for time-varying confounders (e.g., seasonal trends, day-of-week effects) by ensuring that exposures were compared within the same temporal framework. Observations with a mortality count of zero within a given stratum were excluded.

#### Statistical Adjustments for Confounders

To minimize bias in the association between PM_10_ exposure and all-cause mortality, we accounted for confounders by including in the model the four-day moving averages of two key variables, as proposed by Tobias et al. [10]: (i) the 24-h mean temperature from the preceding four days and (ii) the 24-h mean relative humidity from the preceding four days. To account for potential nonlinear relationships, natural cubic B-spline functions were applied. The four-day moving average of temperature was modeled with six degrees of freedom to capture its complex and nonlinear association with mortality, while the four-day moving average of relative humidity was modeled with three degrees of freedom to account for its simpler and more stable relationship, thus avoiding overfitting.

To test the robustness of the current model’s RR estimates to the choice of moving average windows and degrees of freedom, as proposed by Tobias et al. [10], we conducted a sensitivity analysis. This involved systematically varying the moving average time windows from three to seven days and the degrees of freedom from three to six for both temperature and relative humidity, resulting in 400 different adjustment combinations. Detailed information is provided in Supplementary Material 2.

#### Three Different Lag Structures for the Associations between PM_10_ Exposure and All-Cause Mortality

##### Scenario 1: Independent Lag Effects Model

In this model, each lag day was treated as independent, meaning that each was fitted into the model separately, with no assumed interaction effects or influences from neighboring lag days [14]. Each model independently provided an RR estimate for all-cause mortality, with each estimate representing the effect of a specific lag day. We fitted a conditional quasi-Poisson regression model for Scenario 1:

$$\mathit{log}\left(E\left({Y}_{t,s=Dow \times \text{ Month }\times \text{ Year}}\right)\right)=\alpha +ns\left(T\right)+ns\left(RH\right)+{\upbeta }_{l}PM{10}_{l}$$

where ${Y}_{t,s}$ represents daily mortality on day $t$ and is assumed to follow a Poisson distribution with overdispersion (i.e., its variance is not necessarily equal to its mean). The model is conditioned on the total number of deaths in each stratum $s$, defined by the combination of day-of-week, within-month, and year. The terms $ns\left(T\right)$ and $ns\left(RH\right)$ represent natural cubic spline functions of temperature $T$ and relative humidity $RH$, respectively, with degrees of freedom as specified above, accounting for their potential confounding effects on mortality. The term ${\beta }_{l}PM{10}_{l}$ captures the effect of PM_10_ on a specified lag day $l$.

##### Scenario 2: Joint Lag Effects Model

Unlike Scenario 1, this model assumed that lag days may have joint lag effects, creating effects that cannot be easily disentangled. All lag days were modeled together, similar to the multivariable analysis that adjusts confounding effects jointly within a single model described by Tobias et al. [10]. Our model estimated RR for each lag day while simultaneously accounting for potential confounding effects from all other lag days within a single model:

$$\mathit{log}\left(E\left({Y}_{t,s=Dow \times \text{ Month }\times \text{ Year}}\right)\right)=\alpha +ns\left(T\right)+ns\left(RH\right)+{\sum }_{l=0}^{{L}_{max}}{\beta }_{l}PM{10}_{l}$$

where ${L}_{max}$ denotes a pre-specified maximum number of lag days (from 1 to 21 days) to which the health effect—mortality, in this case—could be attributed.

##### Scenario 3: Cumulative Lag Effects Model

We built upon the lag-stratified distributed lag model introduced by Bhaskaran et al. [14], in which effect estimates are grouped (e.g., days 1 and 2 share the same effect, while days 3 to 7 share another effect) based on broad patterns observed in the unconstrained model. However, our cumulative lag effects model extends this approach by explicitly accounting for stepwise cumulative effects over multiple lag days. The conditional quasi-Poisson regression model for Scenario 3 is fitted as:

$$\mathit{log}\left(E\left({Y}_{t,s=Dow \times \text{ Month }\times \text{ Year}}\right)\right)=\alpha +ns\left(T\right)+ns\left(RH\right)+{\sum }_{l=0}^{{L}_{target}}{\beta }_{l}PM{10}_{l}$$

In this model, the estimated RR for a given lag day ${L}_{target}$ is influenced only by the exposures that occur on subsequent days until the health outcome is observed, while earlier exposures (i.e., those before the target lag) are assumed to have no effect on the health outcome. We adopted a stepwise cumulative approach, in which lag day’s variables are incrementally incorporated into the model, allowing the RR for each specific lag to be estimated independently. Unlike Scenario 2, which was constrained by a pre-defined maximum lag ${L}_{max}$, Scenario 3 allowed for a more robust estimation of lag-specific effects.

##### A Unified Framework for Scenarios

**Fig. 1:**
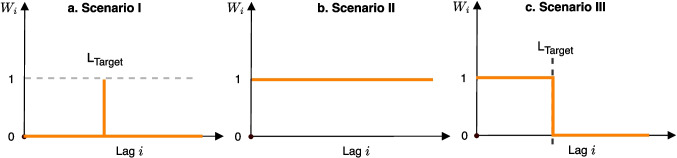
Weighting schemes for Scenarios 1 (**a**), 2 (**b**) and 3 (**c**) within a unified framework. In Scenario 1, only the target lag day is assigned a weight of 1, with all other days set to 0. In Scenario 2, all days up to the maximum lag day receive a weight of 1. In Scenario 3, lag days up to and including the target lag day are assigned a weight of 1, while earlier days receive a weight of 0. In our analyses *i* corresponds to lag days from 0 to 21

More generally, the three scenarios aforementioned can be expressed within a unified framework:

$$\mathit{log}\left(E\left({Y}_{t,s=Dow \times \text{ Month }\times \text{ Year}}\right)\right)=\alpha +ns\left(T\right)+ns\left(RH\right)+{\sum }_{l=0}^{{L}_{target}}{W}_{l}{\beta }_{l}PM{10}_{l}$$

where ${W}_{l}$ represents the weight assigned to each specific lag day. The weighting schemes for ${W}_{l}$ are illustrated in Fig. 1. In Scenario 1, only the target lag day is assigned a weight of 1, with all other days set to 0. In Scenario 2, all days up to the maximum lag day receive a weight of 1. In Scenario 3, lag days up to and including the target lag day are assigned a weight of 1, while earlier days receive a weight of 0. In addition to the weighting schemes discussed above, Supplementary Material 3 presents several additional variants designed to capture more complex lagging effects of environmental exposures. However, a detailed exploration of these is beyond the scope of this paper.

#### Autocorrelation

Environmental exposure variables often exhibit medium-term trends and seasonal variability, resulting in stronger correlations between proximate observations than between those further apart in time. Autocorrelation measures the linear relationship between observations at different lagging time windows to identify underlying trends, seasonality, or persistent patterns in exposure and mortality data; therefore, to assess the extent of temporal correlation in our data, we examined autocorrelations in the time series of daily death counts and PM_10_ exposure at lag values ranging from 0 to 21 days. This ensured that our modeling approach—particularly the different lag structures applied—appropriately accounted for potential time-dependent patterns in the exposure–response relationship.

#### Sensitivity Analysis

To assess the robustness of our results, we conducted a sensitivity analysis by incorporating age groups (below 65 years and 65 or older) as additional confounders into the model for the Valencia dataset to observe their impact on the overall results [20]. A fixed-effects conditional quasi-Poisson regression model was applied to quantify the associations between PM_10_ exposure and all-cause mortality while adjusting for age—in addition to temperature and relative humidity—within the three different lag structures. The London dataset was not stratified by age due to data availability constraints, which made a sensitivity analysis infeasible.

#### Subgroup Analysis

Distinct from the sensitivity analysis, a subgroup analysis was conducted separately for the two age groups (under 65 years and 65 years or older) in the Valencia dataset, treating each age group as an independent dataset. The subgroup analysis investigated whether age modifies the association between PM_10_ exposure and all-cause mortality, thus identifying potential age-specific differences relevant for targeted public health interventions. We applied a fixed-effects conditional quasi-Poisson regression model to quantify the associations between PM_10_ exposure and all-cause mortality, adjusting for temperature and relative humidity, using the three different lag structures. For the London dataset, subgroup analysis was not possible because age-stratified mortality data were unavailable.

### Ethics

This study used publicly available, open-source, anonymized data for secondary analysis. No additional ethical approval was therefore required, and the analysis adhered to ethical research principles by using data with no personal or identifying information. According to the guidelines of the Declaration of Helsinki and international standards for the use of publicly available data, studies employing such data sources are also generally exempt from formal ethical review [21]. This study was reported in accordance with the STrengthening the Reporting of OBservational studies in Epidemiology (STROBE) statement (Supplementary Material 4) [22].

## Results

### Study Population and Relevant Autocorrelation Patterns

**Fig. 2:**
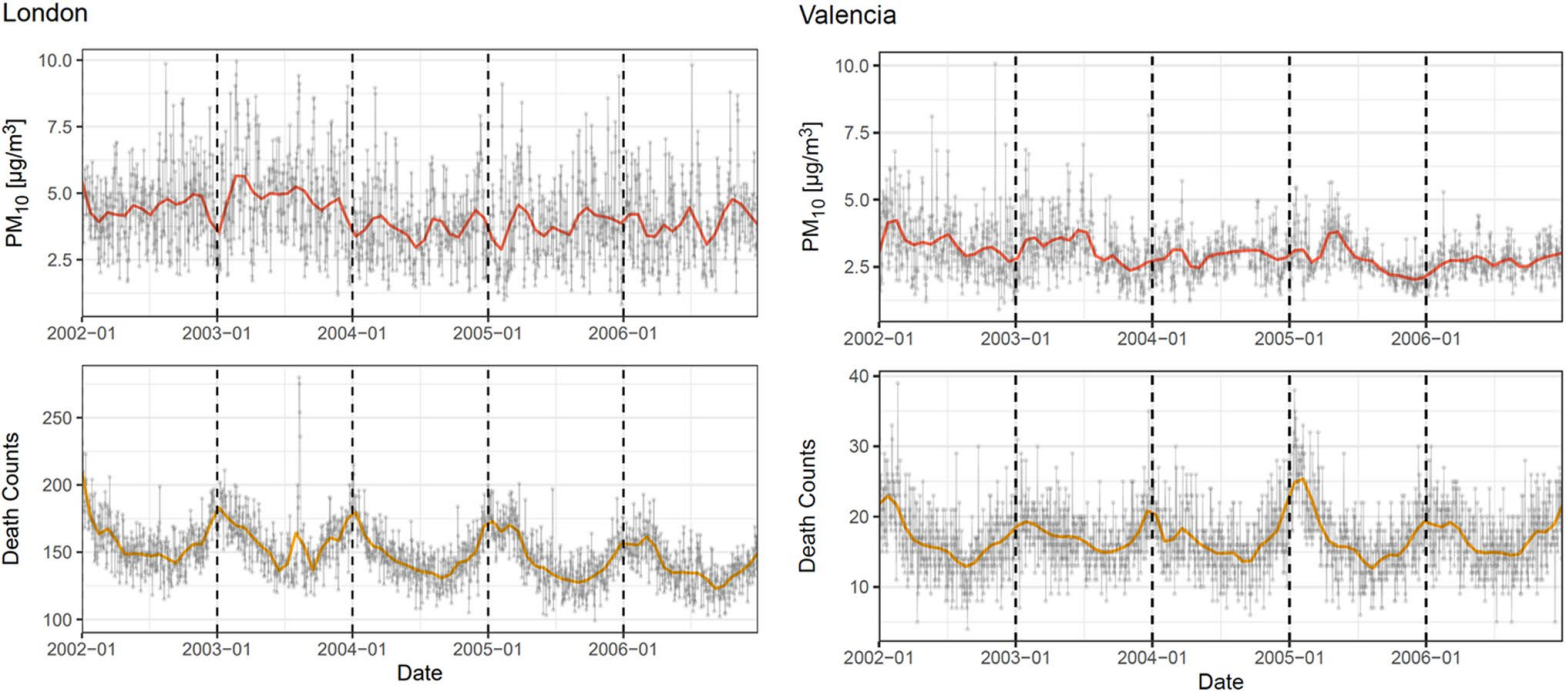
Time series data for all-cause mortality counts and PM_10_ exposure for Valencia and London. The trends for all-cause mortality counts and PM_10_ exposure concentrations, estimated using the B-splines methodology, provide a smooth visualization of the time series over the study period

A total of 30,887 all-cause mortality cases were recorded in Valencia and 273,003 in London from January 1, 2002, to December 31, 2006, spanning 1,826 days. In Valencia, the daily number of all-cause mortality cases ranged from 4 to 39, with a median of 16 and an interquartile range of 14–20. In London, daily deaths ranged from 99 to 280, with a median of 148 and an interquartile range of 135–162. Figure 2 presents the time series data for all-cause mortality counts and PM_10_ exposure concentrations in the two cities. The trends, estimated using the B-splines methodology, provide a smooth visualization of the time series over the study period.

Supplementary Material 5 presents the autocorrelation patterns for all-cause mortality and PM_10_ exposure across a 21-day lagging time window in both cities. For Valencia, there is a sharp drop in PM_10_ exposure autocorrelation values after lag 0, followed by an apparent variation attributable to a pronounced weekly and biweekly periodicity of PM_10_ levels. In contrast, for London, PM_10_ exposure has weaker biweekly periodicity, leading to less variation in the autocorrelation patterns.

For all-cause mortality in London, the autocorrelation values remain above 0.5 during the first seven lag days, gradually declining thereafter and dropping below 0.5. For Valencia, the autocorrelation values remain consistently around 0.25 throughout the analyzed lag period. Both mortality autocorrelation plots indicate no clear weekly or biweekly periodicity.

### Medium-Term Lag-Response Associations for Relative Risk

**Fig. 3:**
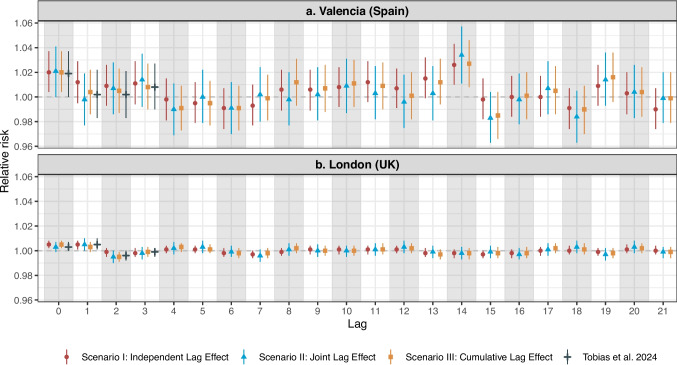
Medium-term lag–response associations of relative risk (lags up to 21 days) for London and Valencia. In this analysis, we accounted for confounders by including in the model the four-day moving averages of two key variables, as proposed by Tobias et al. [10]: (i) the 24-h mean temperature from the preceding four days and (ii) the 24-h mean relative humidity from the preceding four days. Tobias et al. accounted only for relative risk trends up to lag 3 days [10]. Supplementary Material 6 presents the results for relative risks for all-cause mortality and PM_10_ exposure across a 21-day lagging time window in both cities

The overall trends reveal distinct patterns in the RR of PM_10_ exposure concentrations associated with all-cause mortality across lagging time windows up to 21 days. For Valencia, the RR shows considerable fluctuations around 1.00, accompanied by noticeable variability and wider confidence intervals across all lag days (Fig. 3). In contrast, for London, there is a relatively stable pattern over the longer lagging time windows, with the RR consistently close to 1.00, minimal variations, and narrower confidence intervals than for Valencia.

Although our study focused on medium-term lag-response associations—unlike Tobias et al. [10]— our findings for the shorter lagging time windows generally align with that study, particularly in terms of significant relationships. Specifically, for Valencia, a significant association can be seen at lag 0, while significant associations can be observed at both lag 0 and lag 1 for London (Fig. 3 and Supplementary Material 6).

Notably, the Valencia dataset reveals a dynamic temporal pattern. Despite most results being non-significant (Fig. 3), a degree of regularity can be seen. Specifically, from lag 0 to lag 3, the RR starts above 1; from lag 4 to lag 6, it stabilizes below 1, forming a low-level plateau; from lag 7 to lag 14, it rises above 1 again, with a fluctuating upward trend and significance observed at lag 14. Beyond lag 14, the pattern becomes less consistent, with no clear regularity in RR dynamics.

In contrast, the London dataset has a slightly oscillating pattern without strong deviations, with most results being non-significant and the RR oscillating close to 1. Specifically, the RR is significantly greater than 1 at lags 0 and lag 1, drops below 1 at lags 2 and 3, and rises above 1 again at lags 4 and 5. This alternating trend continues but with weaker medium-term effects than in the Valencia dataset and with broader segments: the RR remains below 1 from lags 6 to 8, rises above 1 from lags 9 to 12, and drops below 1 from lags 13 to 16, before increasing again from lags 17 to 21.

**Fig. 4:**
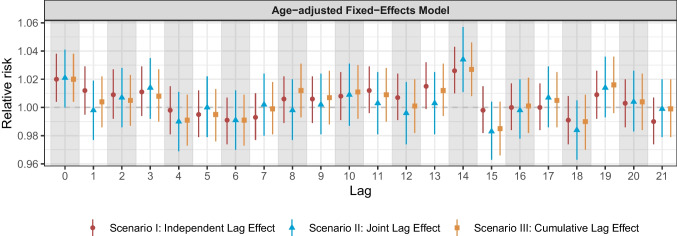
Medium-term lag–response associations of relative risk (lags up to 21 days) for Valencia, adjusted for age, temperature, and relative humidity. To assess the robustness of our results, we conducted a sensitivity analysis by incorporating age groups (below 65 years and 65 or older) as additional confounders into the model for the Valencia dataset to observe their impact on the overall results [20]. A fixed-effects conditional quasi-Poisson regression model was applied to quantify the associations between PM_10_ exposure and all-cause mortality while adjusting for age—in addition to temperature and relative humidity—within the three different lag structures. The London dataset was not stratified by age due to data availability constraints, which made a sensitivity analysis infeasible. Supplementary Material 7 presents the results for the relative risks of all-cause mortality associated with PM_10_ exposure in Valencia, adjusted for age, temperature, and relative humidity

In the sensitivity analysis of the Valencia dataset, no significant differences were seen between the models without age adjustment and those with age included as a confounder. In particular, the general trend of the RR was consistent within the 21-day lagging time window between the two models (Fig. 3a and Fig. 4).

### Different Lag Structures in the Analysis of Exposure–Mortality Relationships

Figure 3 also presents the three different lag methodological structures (Scenarios 1, 2, and 3). These scenarios have somewhat similar trends, but there are variations in the RR values within different lag structures for associations between PM_10_ exposure concentrations and all-cause mortality.

For Valencia, Scenario 2 (joint lag effects model) offers more conservative estimates (lower RR values at most lags) and often has slightly lower RR estimates than Scenario 1 (independent lag effects model) and Scenario 3 (cumulative lag effects model) (Supplementary Material 6). Notably, a peak at lag 14 appears in all three scenarios, indicating a delayed and stronger association between all-cause mortality and PM_10_ exposure 14 days before. In contrast, London dataset not only has a weaker relationship between PM_10_ exposure and all-cause mortality but also exhibits minimal differences across the three scenarios, particularly when compared to the Valencia dataset.

## Discussion

This study addresses a key research gap in environmental epidemiology by extending the research of Tobias et al. and Bhaskaran et al. developing new health insights that were not available from their studies [10, 14]. We analyzed medium-term lag-response associations up to 21 days and went beyond their methodologies, incorporating the independent lag effects model from Bhaskaran et al. [14]., as our Scenario 1; the joint lag effects model from both Bhaskaran et al. and Tobias et al. [10, 14]., as our Scenario 2; and an additional cumulative lag effects model as Scenario 3. This study design allowed us to explore and compare different methodological lag structures in assessing the relationship between PM_10_ exposure and all-cause mortality with medium-term lag-response associations.

**Fig. 5:**
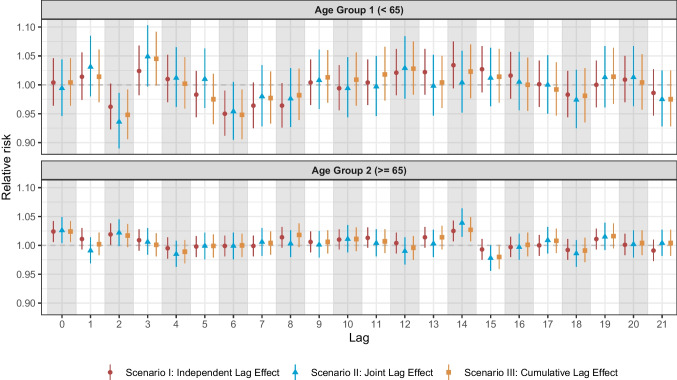
Age-specific subgroup analysis for the Valencia dataset. Distinct from the sensitivity analysis, a subgroup analysis was conducted separately for the two age groups (under 65 years and 65 years or older) in the Valencia dataset, treating each age group as an independent dataset. We applied a fixed-effects conditional quasi-Poisson regression model to quantify the associations between PM_10_ exposure and all-cause mortality, adjusting for temperature and relative humidity, using the three different lag structures. Supplementary Material 8 presents the results for the relative risks of all-cause mortality associated with PM10 exposure from the age-specific subgroup analysis of the Valencia dataset

Consistent with Tobias et al. and other research [23, 24], our results revealed significant immediate and early-time-lag associations between increased all-cause mortality and PM_10_ exposure. This is likely because high PM_10_ exposure concentrations trigger immediate pathophysiological responses, leading to acute respiratory, cardiovascular, and cerebrovascular diseases shortly after pollution spikes [23, 24] (Table 1 and Fig. 3). Elderly populations, especially those with severe comorbidities, are particularly vulnerable to mortality during the early lag stages following air pollution exposure; this was particularly pronounced in the Valencia subgroup analysis, in which we observed a statistically significant association between PM_10_ exposure and all-cause mortality among individuals aged 65 years and older (Fig. 5), with relatively narrow 95% CIs. This is an important public health consideration, and healthcare settings must be aware of the increased risk immediately following PM_10_ exposure. Health authorities, in collaboration with weather forecasting stations, can improve preparedness for a rise in cases by issuing public health alerts, which would enable healthcare facilities to anticipate such periods, implement emergency responses, and manage the expected surge in inpatient admissions.

**Table 1:** Summaries of city-specific differences in vulnerability and public health strategies

Aspect	Valencia	London
Short-term impact (lags 0–3)	Immediate increase in RR, significant at lag 0	Immediate increase in RR, significant at lags 0 and 1
Intermediate trends (lags 4–13)	Alternating trends, possible adaptation, and delayed inflammatory effects	No strong changes; exposure–response relationship remains weak
Delayed impact (lags 14–21)	Significant peak at lag 14	No significant medium-term impact detected
Clinical implications	Requires both immediate and medium-term monitoring for pollution-exposed individuals, with a focus on younger populations	Requires both immediate and medium-term monitoring for pollution-exposed individuals
Policy considerations	Air pollution control should account for both acute and delayed effects	Greater emphasis should be placed on mitigating short-term exposures, followed by addressing delayed effects
Preventive measures	Early interventions for high-risk patients (e.g., medications, air filtration, hospital preparation)	Short-term action plans (e.g., stay-at-home warnings for high-risk groups, hospital preparation)

For both Valencia and London, we observed a pattern in the subsequent lagging-day windows where the RR values dropped below 1 (Fig. 3). The pattern emerged from lag 4 in the Valencia dataset and from lag 2 in the London dataset. Although neither was statistically significant, these decreases are consistent with the “harvesting effect” (mortality displacement) described by Bhaskaran et al. and Schwartz et al. [14, 25], whereby a short-term increase in mortality is followed by a subsequent decrease because the most susceptible individuals have already died.

Population demographic structures may also have impacted the RRs. For London, the relationship between PM_10_ exposure and all-cause mortality was weaker than for Valencia, with narrow 95% CIs, even though the absolute number of all-cause mortality cases in London was greater. This can be explained by demographic differences: in 2006, London's population (median age 34 years) was younger than Valencia's (median age approximately 37.7 years), where 16% were aged 65 and over [26–28]. Specifically, the all-cause mortality attributable to outdoor air pollution differs between these age groups (0.34 deaths per 100,000 inhabitants in 2021 for Great Britain in the 30–34 age group vs. 0.54 deaths per 100,000 in Spain in the 35–39 age group) [29]. Furthermore, Younger people are generally more resilient to PM_10_ exposure than elderly people and have experienced less cumulative exposure over their lifetimes. In contrast, older individuals have experienced longer-term chronic exposure due to their age, increasing their risk of developing chronic diseases and even death. Therefore, it is not surprising that the medium-term lag-response association in London was closer to an RR of 1 than that in Valencia, indicating a weaker exposure–response relationship.

In the Valencia dataset, the RR values had a fluctuating upward trend beyond lag 7, with a decline in RR values at lag 11 (Fig. 3). In contrast, the RR values in London remained relatively stable at around 1. Although these observations were not significant, the pattern noted in Valencia might suggest possible influences from mortality linked to subacute or chronic responses to air pollution or to the progression of chronic conditions, such as chronic obstructive pulmonary disease. Understandably, when comparing peaks of mortality across different lagging time windows, the peak levels from later effects (second peak elevation at lag 10) are noticeably lower than those from the acute and immediate effects (first peak elevations at lag 0 and lag 3) observed shortly after exposure (Fig. 3). Nevertheless, due to the lack of statistical significance, these trends should be interpreted cautiously.

Another interesting result was the significant association between PM_10_ exposure and all-cause mortality at lag 14 in the Valencia dataset, including a similar significant association observed in the subgroup analysis of individuals aged over 65 at the same lag (Fig. 3 and Fig. 5). In contrast, no comparable association was noted in the London dataset. Beyond the influence of Valencia’s older population (Fig. 5), another plausible explanation could be the natural progression of chronic obstructive pulmonary disease. Patients admitted to intensive care units with this disease—especially those with other chronic conditions—may experience delayed health outcomes that peak 2–3 weeks after exposure, likely due to cumulative damage.

A key finding emerged from the subgroup analysis conducted with the Valencia dataset (Fig. 5). Unlike individuals aged 65 and older, those younger than 65 did not exhibit an immediate exposure–mortality relationship at lag 0 but showed initial peaks at lag 3. We also observed an upward trend between lags 7 and 12, followed by a downward trend between lags 13 and 18. Conversely, in the 65 + age group, the RR remained stable and close to 1 throughout a prolonged lagging period from lag 4 to lag 12, followed by a sudden increase thereafter. Although the reasons underlying these differences remain unclear, such temporal patterns may reflect age-related disparities in vulnerability, exposure behaviors, or pre-existing health conditions. Despite the absence of statistical significance, these observations highlight the potential value of implementing age-specific public health messaging following air pollution episodes. This is particularly relevant for individuals with pre-existing respiratory conditions, such as asthma or bronchiectasis, or those at higher risk of pneumonia, who should be advised to wear masks, use air filters, and avoid strenuous outdoor activities during and shortly after periods of high pollution events.

The lag analysis methodologies showed different patterns in the two datasets: there were no obvious differences between the models for London, but there were for Valencia, with Scenario 2 (joint lag effects model) producing more conservative RR estimates (lower RR values at most lags) than the other two scenarios (Supplementary material 6). Scenario 3 (cumulative lag effects model) is conceptually closer to the cumulative progression of health risks associated with prolonged PM_10_ exposure. This model is particularly relevant to vulnerable populations, such as the elderly and people with pre-existing cardiovascular, cerebrovascular, or respiratory conditions, who are more likely to experience cumulative effects from air pollution exposure. While Scenario 3 captures medium-term cumulative exposure patterns and typically produces higher RR estimates compared to Scenario 1, interpreting it as directly capturing specific pathophysiological mechanisms should be done cautiously. Nevertheless, Scenario 3 remains valuable for assessing realistic physiological responses to chronic air pollution exposure, providing important insights into health risks associated with prolonged PM_10_ exposure.

One of the strengths of our study is the scale of the data: large, population-based datasets of registered all-cause mortality cases from two metropolitan cities, spanning 2002 to 2006. This provides a generalizable and representative analysis of medium-term lag-response associations between PM_10_ exposure and all-cause mortality. Additionally, while Bhaskaran et al. considered only a 7-day lag period [14], we extended the lagging time windows to 21 days, offering additional insights into potential residual temporal trends. Furthermore, we demonstrated the robustness of the current model’s RR estimates to the choice of moving average windows and degrees of freedom, as proposed by Tobias et al. [10]. In the sensitivity analysis, by systematically varying the moving average time windows and the degrees of freedom for both temperature and relative humidity, we found that the coefficients of variation are below 0.15%, indicating extremely low variability. This suggests that the RR estimates from different specifications of natural cubic B-splines are tightly clustered around the mean, demonstrating high consistency and stability (Supplementary Material 2).

However, our study has several limitations. First, the all-cause mortality data lacks cause-specific details, which might allow for better recommendations regarding inpatient treatment and healthcare management following acute PM_10_ exposure. Second, our study focuses only on PM_10_ exposure, even though individuals are typically exposed to multiple air pollutants, such as NO_2_ and O_3_, highlighting the need for future research to explore combined pollutant effects. Third, the data spans 2002–2006 and may be considered relatively old; nevertheless, it remains valuable for studying medium-term lag-response associations, and because few environmental epidemiology studies have applied multiple lag methodological structures, our study design represents a novel and methodologically diverse approach in the field. Fourth, although we acknowledge the importance of incorporating PM_2.5_ into the analysis, our assessment of the open-source Air Quality Download Service provided by the European Environment Agency (https://eeadmz1-downloads-webapp.azurewebsites.net/) showed that the available PM_2.5_ data for the selected locations—Valencia, Spain, and London, Great Britain—in the “Historical AirBase data” dataset do not cover the study period of this work. For the Greater London Area, the earliest available PM_2.5_ measurements are from May 15, 2008, while for Valencia, PM_2.5_ data are first available from January 1, 2009.

For future studies, it would be important to define the lag structure a priori based on clinical knowledge of disease evolution (natural history of the disease). For example, decompensations resulting from co-infections induced by increased contamination may be more likely to occur after approximately 15 days of exposure, whereas clinical stroke often presents in an acute form within lags 0–3. This distinction warrants further investigation.

In conclusion, the current study not only highlights the critical importance of addressing medium-term lag-response associations and methodological variations in environmental epidemiology but also has strong clinical and public health implications. By analyzing the temporal dynamics between PM_10_ exposure and all-cause mortality, we provide insights to inform clinical practice and public health strategies. Integrating medium-term lag-response data into public health policies can enhance early-warning systems and adaptive interventions, thus helping to protect vulnerable populations by enabling real-time risk prediction, resource allocation, and proactive healthcare responses. Clinicians can play a vital role by incorporating air quality indices into care workflows, advising patients on preventive measures, and adjusting treatment plans for high-risk groups during pollution spikes. Additionally, public health authorities and healthcare systems can use these findings to better prepare for surges in hospital admissions during identified high-risk periods, mitigating the risks and improving population health outcomes.

## Supplementary Information

Below is the link to the electronic supplementary material.

Supplementary file1 (DOCX 273 KB)

## Data Availability

No datasets were generated or analysed during the current study. The original codes for the current analysis can be found via the Zenodo repository (10.5281/zenodo.16872159).
