# Supplementary material for: Medium-Term Lag-Response Associations Between PM10 Exposure and All-Cause Mortality in Valencia and London: A Time-Stratified Case-Crossover Study
Source: J Epidemiol Glob Health. 2025 Aug 30;15(1):113. doi: 10.1007/s44197-025-00459-x (PMC12398449; doi:10.1007/s44197-025-00459-x)
Supplement: Supplementary file 1 — Supplementary file1 (DOCX 273 KB) [file 44197_2025_459_MOESM1_ESM.docx]

**Title: Medium-Term Lag-Response Associations Between PM_10_ Exposure and All-Cause Mortality in Valencia and London: A Time-Stratified Case-Crossover Study**

**SUPPLEMENTARY MATERIALS**

**Supplementary Material 1.** R codes for three different lag structures for the associations between PM10 exposure and all-cause mortality

**Supplementary Material 2.** Mean relative risks (RR) for all-cause mortality associated with PM₁₀ exposure over a 21-day lag period, averaged across models with varying specifications of natural cubic splines—specifically different moving average windows (N days) and degrees of freedom—for adjusting temperature and relative humidity. The standard deviation (SD) reflects the dispersion of RRs across 400 model runs, while the coefficient of variation (CV), calculated as the ratio of SD to the mean and expressed as a percentage [%], indicates the variability of the estimates. The relatively low CV values suggest that the RR estimates are stable and that variations in spline settings (moving average windows and degrees of freedom) have a minimal impact on the results.

**Supplementary Material 3.** Four additional weighting schemes to capture more complex lag structures of environmental exposures: a) Cascading scheme: Weights are assigned in a stepwise, cascading pattern across lag days. b) Linear declining scheme: Weights decrease linearly from the present day toward earlier lag days. c) Exponential declining scheme: Weights decrease exponentially from the day of exposure to preceding days, suitable for exposures with acute health effects. d) Inverted U-shaped scheme: Weights increase up to a certain lag day and then decline, reflecting exposures with delayed effects mediated by time-dependent biological processes, such as metabolic activation, where adverse health outcomes emerge only after the exposure has undergone biotransformation within the body.

**Supplementary Material 4.** STROBE Statement—Checklist of items that should be included in reports of cohort studies

**Supplementary Material 5.** Autocorrelation for all-cause mortality and PM_10_ exposure across a 21-day lagging time window in both cities **Supplementary Material 6.** Results for relative risks for all-cause mortality and PM_10_ exposure across a 21-day lagging time window in both cities

**Supplementary Material 7.** Results for relative risks for all-cause mortality and PM_10_ exposure across a 21-day lagging time window for Valencia, adjusted for age

**Supplementary Material 8.** Relative risks for all-cause mortality and PM10 exposure across a 21-day lagging time window for Valencia based on subgroups Age Group 1 (< 65) and Age Group 2 (≥ 65)

**Supplementary Material 1. R codes for three different lag structures for the associations between PM_10_ exposure and all-cause mortality**

**Scenario 1: Independent lag effects model**

# results for scenario I

rr.s01 <- list()

for (city in cities) {

data <- mdat[[city]]

rr.s01[[city]] <- lapply(0:lag_max, function(lag) {

# PM10 lag,

var = paste0("pm10_l", lag)

# Each lag day is regressed separately

iformula <- sprintf('all ~ ns.tmean + ns.rh + %s', var) %>%

formula()

# conditioned Poisson regression

model.cc <- gnm(

iformula,

data = data,

family = quasipoisson,

subset = ind > 0,

eliminate = stratum

)

## RR for each lag

res = Epi::ci.exp(model.cc, subset = var) %>%

round(3) %>%

data.table() %>%

set_names(c('Relative risk', 'lower', 'upper')) %>%

mutate(`95% CI` = sprintf('%.3f-%.3f', lower, upper),

lag = lag) %>%

.[, c(5, 1:4)]

}) %>%

bind_rows()

}

**Scenario 2: Joint lag effects model**

# results for scenario II

rr.s02 <- list()

for (city in cities){

data <- mdat[[city]]

# all lag days till the maximum lag are integrated into the model simultaneously.

vars = paste0("pm10_l",0:lag_max, collapse = '+')

iformula <- sprintf('all ~ ns.tmean + ns.rh + %s',vars) %>%

formula()

model.cc <- gnm(

iformula,

data = data,

family = quasipoisson,

subset = ind > 0,

eliminate = stratum)

## RR for each lag

rr.s02[[city]] <- lapply(0:lag_max,function(lag){

var=paste0('pm10_l',lag)

res= Epi::ci.exp(model.cc, subset = var) %>%

round(3) %>%

data.table() %>%

slice(1) %>% # as the "subset" in Epi::ci.exp() use grep to select matched variables, keep the first matched result.

set_names(c('Relative risk','lower','upper')) %>%

mutate(`95% CI`= sprintf('%.3f-%.3f',lower,upper),

lag=lag) %>%

.[,c(5,1:4)]

}) %>%

bind_rows()

}

**Scenario 3: Step-wise lag effects model**

# results for scenario III

rr.s03 <- list()

for (city in cities){

data <- mdat[[city]]

rr.s03[[city]] <- lapply(0:lag_max, function(lag) {

# target lag day

var = paste0("pm10_l", lag)

# lags considered in the regression model

vars = paste0("pm10_l", 0:lag, collapse = '+')

iformula <- sprintf('all ~ ns.tmean + ns.rh + %s', vars) %>%

formula()

model.cc <- gnm(

iformula,

data = data,

family = quasipoisson,

subset = ind > 0,

eliminate = stratum

)

res= Epi::ci.exp(model.cc, subset = var) %>%

round(3) %>%

data.table() %>%

set_names(c('Relative risk','lower','upper')) %>%

mutate(`95% CI`= sprintf('%.3f-%.3f',lower,upper),

lag=lag) %>%

.[,c(5,1:4)]

return(res)

}) %>% bind_rows()

}

**Supplementary Material 2. Mean relative risks (RR) for all-cause mortality associated with PM₁₀ exposure over a 21-day lag period, averaged across models with varying specifications of natural cubic splines—specifically different moving average windows (N days) and degrees of freedom—for adjusting temperature and relative humidity. The standard deviation (SD) reflects the dispersion of RRs across 400 model runs, while the coefficient of variation (CV), calculated as the ratio of SD to the mean and expressed as a percentage [%], indicates the variability of the estimates. The relatively low CV values suggest that the RR estimates are stable and that variations in spline settings (moving average windows and degrees of freedom) have a minimal impact on the results.**

**Table a. The results of coefficient of variation**

|  | | Scenario I | | | Scenario II | | | Scenario III | | |
| --- | --- | --- | --- | --- | --- | --- | --- | --- | --- | --- |
| City | Lag | RR | SD | CV [%] | RR | SD | CV [%] | RR | SD | CV [%] |
| Valencia | 0 | 1.020 | 0.0008 | 0.074 | 1.021 | 0.0008 | 0.081 | 1.020 | 0.0008 | 0.074 |
|  | 1 | 1.013 | 0.0009 | 0.086 | 0.998 | 0.0004 | 0.040 | 1.005 | 0.0007 | 0.066 |
|  | 2 | 1.012 | 0.0011 | 0.110 | 1.008 | 0.0004 | 0.044 | 1.007 | 0.0009 | 0.090 |
|  | 3 | 1.013 | 0.0013 | 0.124 | 1.015 | 0.0008 | 0.081 | 1.009 | 0.0009 | 0.089 |
|  | 4 | 0.998 | 0.0011 | 0.107 | 0.990 | 0.0005 | 0.054 | 0.991 | 0.0008 | 0.080 |
|  | 5 | 0.996 | 0.0011 | 0.108 | 1.001 | 0.0006 | 0.060 | 0.995 | 0.0008 | 0.078 |
|  | 6 | 0.991 | 0.0009 | 0.092 | 0.991 | 0.0005 | 0.046 | 0.991 | 0.0007 | 0.067 |
|  | 7 | 0.993 | 0.0008 | 0.078 | 1.002 | 0.0006 | 0.064 | 0.999 | 0.0006 | 0.062 |
|  | 8 | 1.006 | 0.0007 | 0.066 | 0.998 | 0.0003 | 0.031 | 1.012 | 0.0005 | 0.047 |
|  | 9 | 1.006 | 0.0005 | 0.053 | 1.003 | 0.0004 | 0.040 | 1.008 | 0.0005 | 0.049 |
|  | 10 | 1.008 | 0.0005 | 0.051 | 1.009 | 0.0005 | 0.047 | 1.011 | 0.0005 | 0.045 |
|  | 11 | 1.012 | 0.0005 | 0.049 | 1.002 | 0.0005 | 0.050 | 1.009 | 0.0005 | 0.049 |
|  | 12 | 1.007 | 0.0005 | 0.047 | 0.996 | 0.0004 | 0.040 | 1.001 | 0.0004 | 0.041 |
|  | 13 | 1.016 | 0.0006 | 0.060 | 1.003 | 0.0005 | 0.052 | 1.013 | 0.0006 | 0.058 |
|  | 14 | 1.026 | 0.0007 | 0.069 | 1.034 | 0.0005 | 0.046 | 1.027 | 0.0006 | 0.057 |
|  | 15 | 0.998 | 0.0007 | 0.069 | 0.983 | 0.0005 | 0.049 | 0.984 | 0.0005 | 0.050 |
|  | 16 | 0.999 | 0.0006 | 0.064 | 0.998 | 0.0002 | 0.021 | 1.001 | 0.0004 | 0.039 |
|  | 17 | 1.000 | 0.0006 | 0.057 | 1.007 | 0.0004 | 0.039 | 1.005 | 0.0004 | 0.036 |
|  | 18 | 0.991 | 0.0005 | 0.055 | 0.984 | 0.0000 | 0.000 | 0.990 | 0.0004 | 0.038 |
|  | 19 | 1.009 | 0.0006 | 0.060 | 1.015 | 0.0005 | 0.050 | 1.016 | 0.0006 | 0.059 |
|  | 20 | 1.003 | 0.0006 | 0.055 | 1.005 | 0.0005 | 0.050 | 1.005 | 0.0005 | 0.049 |
|  | 21 | 0.990 | 0.0006 | 0.056 | 0.999 | 0.0006 | 0.060 | 0.999 | 0.0006 | 0.060 |
| London | 0 | 1.007 | 0.0010 | 0.100 | 1.004 | 0.0008 | 0.081 | 1.007 | 0.0010 | 0.100 |
|  | 1 | 1.006 | 0.0010 | 0.096 | 1.006 | 0.0005 | 0.054 | 1.004 | 0.0006 | 0.059 |
|  | 2 | 1.000 | 0.0010 | 0.101 | 0.995 | 0.0005 | 0.050 | 0.996 | 0.0006 | 0.065 |
|  | 3 | 0.999 | 0.0008 | 0.081 | 0.998 | 0.0002 | 0.019 | 0.998 | 0.0005 | 0.051 |
|  | 4 | 1.001 | 0.0007 | 0.066 | 1.002 | 0.0005 | 0.049 | 1.002 | 0.0006 | 0.060 |
|  | 5 | 1.001 | 0.0006 | 0.055 | 1.003 | 0.0005 | 0.049 | 1.001 | 0.0005 | 0.050 |
|  | 6 | 0.998 | 0.0005 | 0.049 | 0.999 | 0.0003 | 0.033 | 0.998 | 0.0004 | 0.042 |
|  | 7 | 0.997 | 0.0006 | 0.056 | 0.996 | 0.0005 | 0.047 | 0.998 | 0.0000 | 0.000 |
|  | 8 | 0.999 | 0.0005 | 0.047 | 1.001 | 0.0000 | 0.000 | 1.001 | 0.0005 | 0.049 |
|  | 9 | 1.000 | 0.0002 | 0.024 | 1.000 | 0.0000 | 0.000 | 1.000 | 0.0001 | 0.013 |
|  | 10 | 1.000 | 0.0003 | 0.034 | 0.999 | 0.0005 | 0.049 | 1.000 | 0.0000 | 0.005 |
|  | 11 | 1.000 | 0.0004 | 0.035 | 1.001 | 0.0002 | 0.020 | 1.001 | 0.0003 | 0.028 |
|  | 12 | 1.000 | 0.0005 | 0.049 | 1.002 | 0.0005 | 0.049 | 1.002 | 0.0000 | 0.000 |
|  | 13 | 0.998 | 0.0002 | 0.022 | 0.999 | 0.0000 | 0.000 | 0.997 | 0.0003 | 0.026 |
|  | 14 | 0.998 | 0.0005 | 0.049 | 0.998 | 0.0005 | 0.046 | 0.998 | 0.0005 | 0.054 |
|  | 15 | 0.997 | 0.0004 | 0.041 | 1.000 | 0.0005 | 0.050 | 0.999 | 0.0005 | 0.047 |
|  | 16 | 0.998 | 0.0005 | 0.047 | 0.998 | 0.0005 | 0.049 | 0.998 | 0.0005 | 0.050 |
|  | 17 | 1.000 | 0.0004 | 0.044 | 1.001 | 0.0004 | 0.042 | 1.002 | 0.0004 | 0.038 |
|  | 18 | 1.000 | 0.0005 | 0.047 | 1.003 | 0.0004 | 0.044 | 1.001 | 0.0003 | 0.034 |
|  | 19 | 0.999 | 0.0003 | 0.029 | 0.997 | 0.0005 | 0.049 | 0.998 | 0.0003 | 0.034 |
|  | 20 | 1.001 | 0.0005 | 0.047 | 1.003 | 0.0004 | 0.036 | 1.002 | 0.0002 | 0.020 |
|  | 21 | 1.000 | 0.0005 | 0.050 | 0.999 | 0.0004 | 0.037 | 0.999 | 0.0004 | 0.037 |

**Fig a. Coefficients of variation of relative risk estimates from models of different scenarios based on varying temperature and humidity adjustments, generated using different specifications of moving average windows and degrees of freedom in natural cubic B-splines.**

**Description**

Following Tobias et al., we incorporated 24-hour mean temperature and relative humidity from the preceding four days into natural cubic B-spline functions with six and three degrees of freedom, respectively, to adjust for potential confounding effects. To assess the sensitivity of relative risk (RR) estimates to the choice of *moving average windows* and *degrees of freedom*, we systematically varied the moving average windows from 3 to 7 days and the degrees of freedom from 3 to 6 for both temperature and relative humidity. This resulted in 5 × 4 × 5 × 4 = 400 different combinations. For each combination, RR was estimated using the respective adjusted temperature and humidity values in models of different scenarios.

To quantify the variability across these 400 RR estimates, we computed the standard deviation (SD) and the coefficient of variation (CV), where CV is defined as the ratio of the SD to the mean, multiplied by 100 to express it as a percentage. The CV serves as an indicator of the robustness of RR estimates to variations in spline parameter settings.

As shown in Fig a. and Table a., the coefficients of variation are below 0.15%, indicating extremely low variability. This suggests that the relative risk estimates from different specifications of natural cubic B-splines are tightly clustered around the mean, demonstrating high consistency and stability.

**Supplementary Material 3.** Four additional weighting schemes to capture more complex lag structures of environmental exposures: a) Cascading scheme: Weights are assigned in a stepwise, cascading pattern across lag days. b) Linear declining scheme: Weights decrease linearly from the present day toward earlier lag days. c) Exponential declining scheme: Weights decrease exponentially from the day of exposure to preceding days, suitable for exposures with acute health effects. d) Inverted U-shaped scheme: Weights increase up to a certain lag day and then decline, reflecting exposures with delayed effects mediated by time-dependent biological processes, such as metabolic activation, where adverse health outcomes emerge only after the exposure has undergone biotransformation within the body.

**
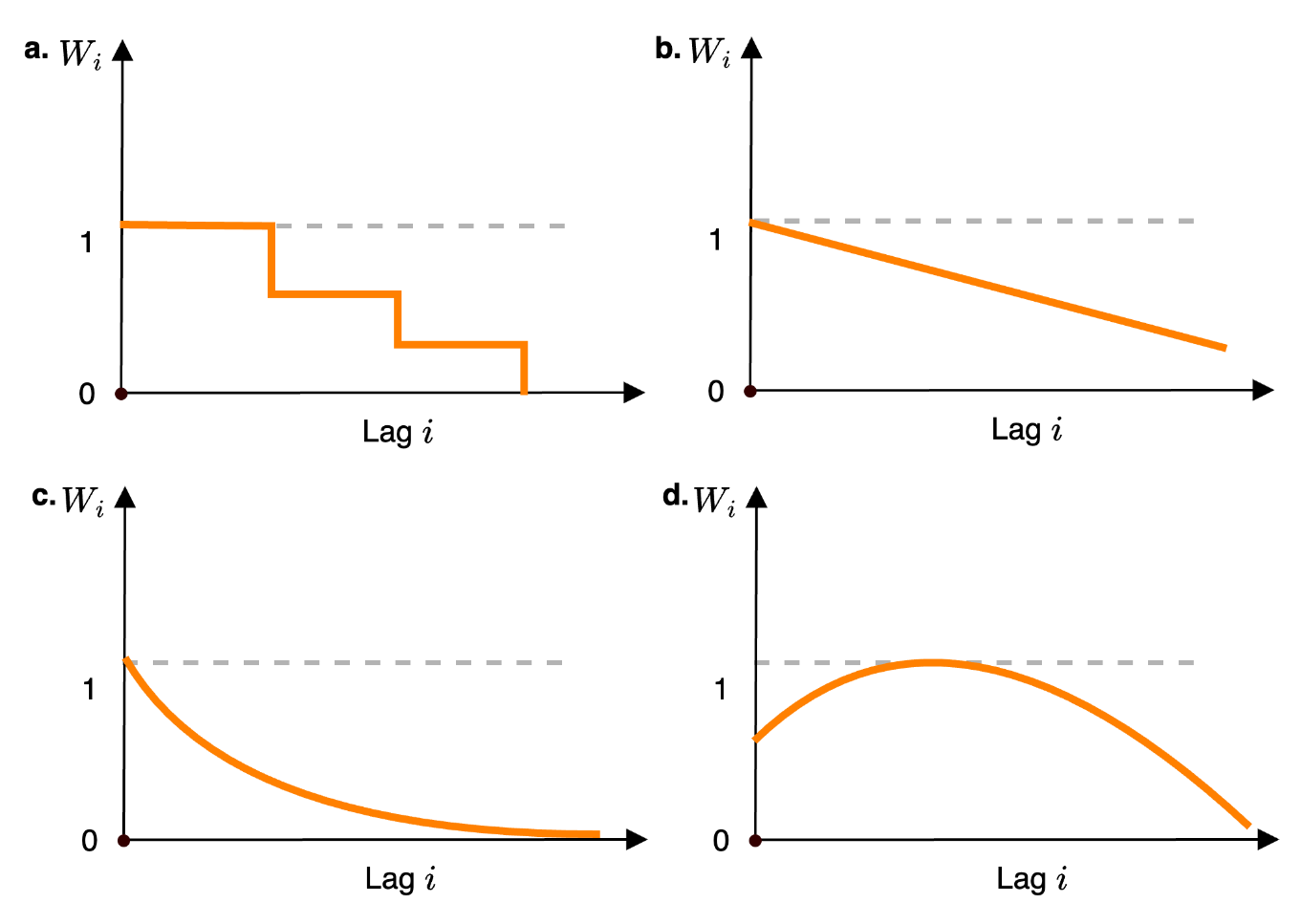
**

**Supplementary Material 4. STROBE Statement—Checklist of items that should be included in reports of cohort studies**

Study title: Medium-Term Lag-Response Associations Between PM10 Exposure and All-Cause Mortality in Valencia and London: A Time-Stratified Case-Crossover Study

|  | **Item No** | **Recommendation** | **Page(s)** |
| --- | --- | --- | --- |
| **Title and abstract** | 1 | (*a*) Indicate the study’s design with a commonly used term in the title or the abstract | 1-3 |
|  |  | (*b*) Provide in the abstract an informative and balanced summary of what was done and what was found | 3 |
| Introduction | | |  |
| Background/rationale | 2 | Explain the scientific background and rationale for the investigation being reported | 4-5 |
| Objectives | 3 | State specific objectives, including any prespecified hypotheses | 6 |
| Methods | | |  |
| Study design | 4 | Present key elements of study design early in the paper | 7 |
| Setting | 5 | Describe the setting, locations, and relevant dates, including periods of recruitment, exposure, follow-up, and data collection | 7 |
| Participants | 6 | (*a*) Give the eligibility criteria, and the sources and methods of selection of participants. Describe methods of follow-up | 7 |
|  |  | (*b*) For matched studies, give matching criteria and number of exposed and unexposed | 8, our study compared with cases days with control days. |
| Variables | 7 | Clearly define all outcomes, exposures, predictors, potential confounders, and effect modifiers. Give diagnostic criteria, if applicable | 7-9 |
| Data sources/ measurement | 8 | For each variable of interest, give sources of data and details of methods of assessment (measurement). Describe comparability of assessment methods if there is more than one group | 7, As we secondarily analyzed the data, we described the sources from which the data originated. |
| Bias | 9 | Describe any efforts to address potential sources of bias | n/a |
| Study size | 10 | Explain how the study size was arrived at | 7, We included all available data on daily all-cause mortality counts and environmental exposure from 2002 to 2006. No data were excluded. |
| Quantitative variables | 11 | Explain how quantitative variables were handled in the analyses. If applicable, describe which groupings were chosen and why | 8-10 |
| Statistical methods | 12 | (*a*) Describe all statistical methods, including those used to control for confounding | 8-10 |
|  |  | (*b*) Describe any methods used to examine subgroups and interactions | 11 |
|  |  | (*c*) Explain how missing data were addressed | There were no missing data, as documented. |
|  |  | (*d*) If applicable, explain how loss to follow-up was addressed | n/a |
|  |  | (*e*) Describe any sensitivity analyses | 11 |
| Results | | |  |
| Participants | 13 | (a) Report numbers of individuals at each stage of study—e.g., numbers potentially eligible, examined for eligibility, confirmed eligible, included in the study, completing follow-up, and analysed | 13 |
|  |  | (b) Give reasons for non-participation at each stage | n/a |
|  |  | (c) Consider use of a flow diagram | n/a |
| Descriptive data | 14 | (a) Give characteristics of study participants (e.g., demographic, clinical, social) and information on exposures and potential confounders | 13 |
|  |  | (b) Indicate number of participants with missing data for each variable of interest | n/a |
|  |  | (c) Summarise follow-up time (e.g., average and total amount) | n/a |
| Outcome data | 15 | Report numbers of outcome events or summary measures over time | 13-15 |
| Main results | 16 | (*a*) Give unadjusted estimates and, if applicable, confounder-adjusted estimates and their precision (e.g., 95% confidence interval). Make clear which confounders were adjusted for and why they were included | 13-15, Figure 3, and Suplementary material 6 |
|  |  | (*b*) Report category boundaries when continuous variables were categorized | 13, we reported an interquartile range for mortality cases for both cities |
|  |  | (*c*) If relevant, consider translating estimates of relative risk into absolute risk for a meaningful time period | 13-15, Figure 3, and Suplementary material 6 |
| Other analyses | 17 | Report other analyses done—e.g., analyses of subgroups and interactions, and sensitivity analyses | 14, Figure 4 and Figure 5 |
| Discussion | | |  |
| Key results | 18 | Summarise key results with reference to study objectives | 16-18 |
| Limitations | 19 | Discuss limitations of the study, taking into account sources of potential bias or imprecision. Discuss both direction and magnitude of any potential bias | 19 |
| Interpretation | 20 | Give a cautious overall interpretation of results considering objectives, limitations, multiplicity of analyses, results from similar studies, and other relevant evidence | 16-19 |
| Generalisability | 21 | Discuss the generalisability (external validity) of the study results | 19, we reported the generalization and representativeness in the strengths of the study. |
| Other information | | |  |
| Funding | 22 | Give the source of funding and the role of the funders for the present study and, if applicable, for the original study on which the present article is based | 21 |

**Supplementary Material 5. Autocorrelation for all-cause mortality and PM_10_ exposure across a 21-day lagging time window in both cities**


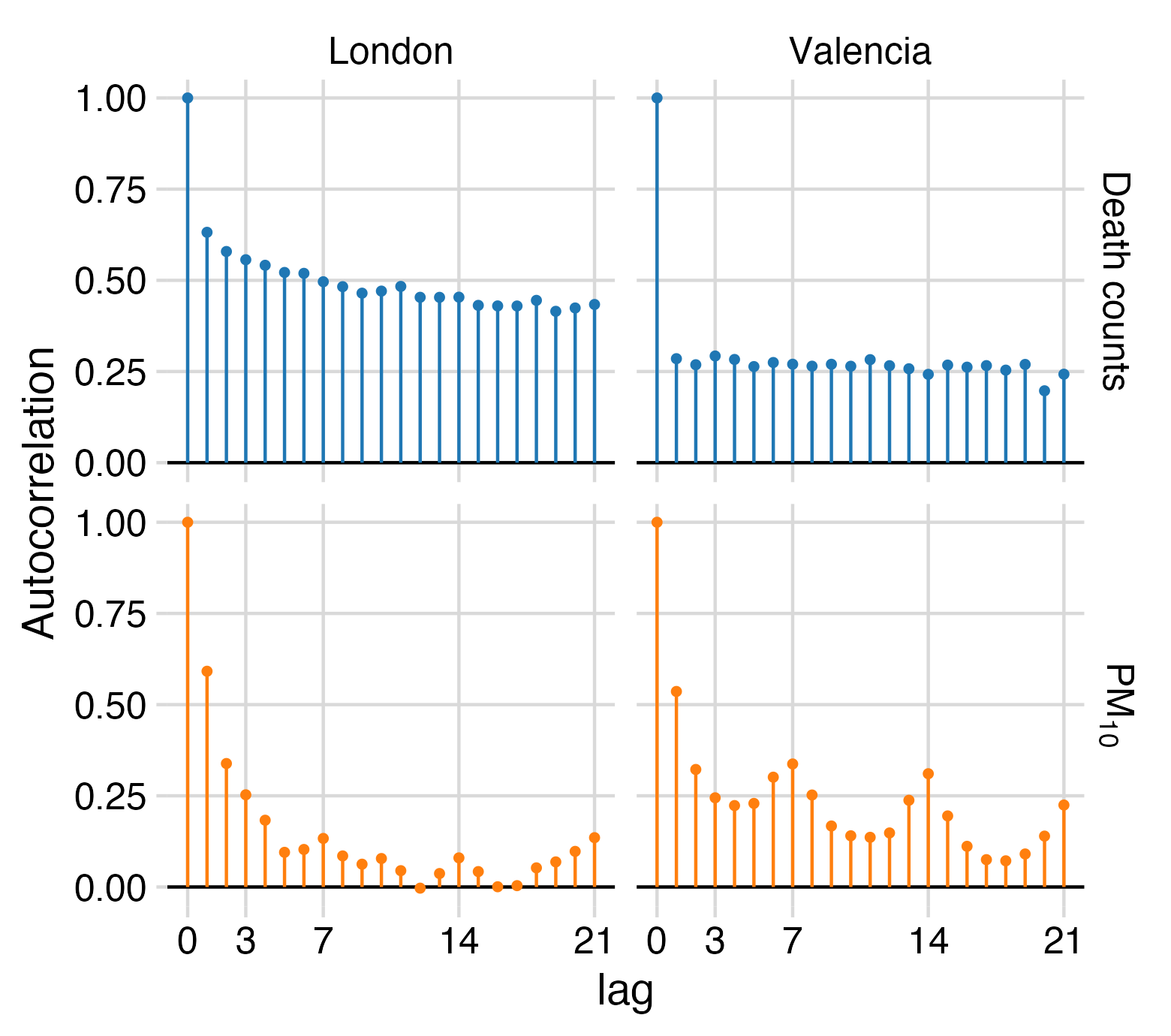


**Supplementary Material 6. Results for relative risks for all-cause mortality and PM_10_ exposure across a 21-day lagging time window in both cities**

| city | Lag | Scenario I | Scenario II | Scenario III | Tobias et al. 2024 |
| --- | --- | --- | --- | --- | --- |
| **Valencia** | 0 | 1.020 [1.004-1.037] | 1.021 [1.000-1.041] | 1.020 [1.004-1.037] | 1.019 [1.000-1.037] |
|  | 1 | 1.012 [0.995-1.029] | 0.998 [0.977-1.019] | 1.004 [0.986-1.022] | 1.002 [0.983-1.022] |
|  | 2 | 1.009 [0.993-1.026] | 1.007 [0.986-1.028] | 1.005 [0.987-1.023] | 1.002 [0.983-1.021] |
|  | 3 | 1.011 [0.994-1.029] | 1.014 [0.992-1.035] | 1.008 [0.990-1.027] | 1.008 [0.990-1.027] |
|  | 4 | 0.998 [0.981-1.015] | 0.990 [0.969-1.011] | 0.991 [0.973-1.009] | n/a |
|  | 5 | 0.995 [0.979-1.012] | 1.000 [0.979-1.022] | 0.995 [0.977-1.013] | n/a |
|  | 6 | 0.991 [0.974-1.007] | 0.991 [0.970-1.012] | 0.991 [0.973-1.009] | n/a |
|  | 7 | 0.993 [0.977-1.010] | 1.002 [0.980-1.024] | 0.999 [0.981-1.018] | n/a |
|  | 8 | 1.006 [0.989-1.022] | 0.998 [0.977-1.020] | 1.012 [0.993-1.031] | n/a |
|  | 9 | 1.006 [0.989-1.022] | 1.002 [0.981-1.024] | 1.007 [0.988-1.026] | n/a |
|  | 10 | 1.008 [0.992-1.024] | 1.009 [0.987-1.031] | 1.011 [0.992-1.030] | n/a |
|  | 11 | 1.012 [0.996-1.029] | 1.003 [0.982-1.025] | 1.009 [0.990-1.028] | n/a |
|  | 12 | 1.007 [0.991-1.023] | 0.996 [0.975-1.018] | 1.001 [0.982-1.020] | n/a |
|  | 13 | 1.015 [0.999-1.032] | 1.003 [0.981-1.025] | 1.012 [0.994-1.031] | n/a |
|  | 14 | 1.026 [1.010-1.043] | 1.034 [1.011-1.057] | 1.027 [1.008-1.046] | n/a |
|  | 15 | 0.998 [0.982-1.015] | 0.983 [0.963-1.004] | 0.985 [0.966-1.004] | n/a |
|  | 16 | 1.000 [0.984-1.017] | 0.998 [0.978-1.019] | 1.001 [0.982-1.020] | n/a |
|  | 17 | 1.000 [0.984-1.017] | 1.007 [0.986-1.029] | 1.005 [0.986-1.025] | n/a |
|  | 18 | 0.991 [0.974-1.007] | 0.984 [0.963-1.005] | 0.990 [0.970-1.009] | n/a |
|  | 19 | 1.009 [0.993-1.026] | 1.014 [0.993-1.036] | 1.016 [0.996-1.036] | n/a |
|  | 20 | 1.003 [0.986-1.020] | 1.004 [0.983-1.026] | 1.004 [0.984-1.024] | n/a |
|  | 21 | 0.990 [0.974-1.007] | 0.999 [0.979-1.020] | 0.999 [0.979-1.020] | n/a |
| **London** | 0 | 1.005 [1.002-1.008] | 1.003 [0.999-1.007] | 1.005 [1.002-1.008] | 1.003 [1.000-1.007] |
|  | 1 | 1.005 [1.001-1.008] | 1.005 [1.000-1.010] | 1.003 [0.999-1.007] | 1.005 [1.001-1.010] |
|  | 2 | 0.999 [0.995-1.002] | 0.995 [0.990-1.000] | 0.995 [0.991-0.999] | 0.996 [0.992-1.000] |
|  | 3 | 0.998 [0.995-1.002] | 0.998 [0.993-1.003] | 0.999 [0.995-1.003] | 0.999 [0.995-1.002] |
|  | 4 | 1.001 [0.998-1.004] | 1.002 [0.997-1.007] | 1.003 [0.999-1.006] | n/a |
|  | 5 | 1.001 [0.998-1.004] | 1.003 [0.998-1.008] | 1.001 [0.998-1.005] | n/a |
|  | 6 | 0.998 [0.995-1.002] | 0.999 [0.995-1.004] | 0.998 [0.994-1.002] | n/a |
|  | 7 | 0.997 [0.994-1.000] | 0.996 [0.991-1.001] | 0.998 [0.994-1.002] | n/a |
|  | 8 | 0.999 [0.996-1.002] | 1.001 [0.996-1.006] | 1.002 [0.998-1.006] | n/a |
|  | 9 | 1.001 [0.997-1.004] | 1.000 [0.995-1.005] | 1.000 [0.996-1.004] | n/a |
|  | 10 | 1.001 [0.997-1.004] | 1.000 [0.995-1.004] | 1.000 [0.996-1.004] | n/a |
|  | 11 | 1.001 [0.997-1.004] | 1.001 [0.996-1.006] | 1.001 [0.997-1.006] | n/a |
|  | 12 | 1.001 [0.998-1.004] | 1.003 [0.998-1.008] | 1.002 [0.998-1.006] | n/a |
|  | 13 | 0.998 [0.995-1.002] | 0.999 [0.994-1.004] | 0.997 [0.993-1.001] | n/a |
|  | 14 | 0.998 [0.994-1.001] | 0.998 [0.993-1.003] | 0.998 [0.993-1.002] | n/a |
|  | 15 | 0.997 [0.994-1.000] | 0.999 [0.994-1.004] | 0.998 [0.994-1.003] | n/a |
|  | 16 | 0.998 [0.994-1.001] | 0.997 [0.993-1.002] | 0.998 [0.994-1.003] | n/a |
|  | 17 | 1.000 [0.996-1.003] | 1.001 [0.996-1.006] | 1.002 [0.998-1.006] | n/a |
|  | 18 | 1.000 [0.997-1.004] | 1.003 [0.998-1.008] | 1.001 [0.997-1.006] | n/a |
|  | 19 | 0.999 [0.996-1.002] | 0.997 [0.992-1.002] | 0.998 [0.994-1.002] | n/a |
|  | 20 | 1.001 [0.998-1.005] | 1.003 [0.998-1.008] | 1.002 [0.998-1.006] | n/a |
|  | 21 | 1.000 [0.997-1.004] | 0.999 [0.994-1.003] | 0.999 [0.994-1.003] | n/a |

**Note: The results correspond to Figure 3.**

**Supplementary Material 7. Results for relative risks for all-cause mortality and PM_10_ exposure across a 21-day lagging time window for Valencia, adjusted for age, temperature, and relative humidity**

| Lag | Scenario I | Scenario II | Scenario III |
| --- | --- | --- | --- |
| 0 | 1.020 [1.004-1.038] | 1.021 [1.000-1.041] | 1.020 [1.004-1.038] |
| 1 | 1.012 [0.995-1.029] | 0.998 [0.977-1.019] | 1.004 [0.986-1.022] |
| 2 | 1.009 [0.992-1.027] | 1.007 [0.986-1.028] | 1.005 [0.987-1.023] |
| 3 | 1.011 [0.994-1.029] | 1.014 [0.992-1.035] | 1.008 [0.990-1.027] |
| 4 | 0.998 [0.981-1.015] | 0.990 [0.969-1.011] | 0.991 [0.973-1.009] |
| 5 | 0.995 [0.979-1.012] | 1.000 [0.979-1.022] | 0.995 [0.976-1.013] |
| 6 | 0.991 [0.974-1.007] | 0.991 [0.970-1.012] | 0.991 [0.973-1.009] |
| 7 | 0.993 [0.977-1.010] | 1.002 [0.980-1.024] | 0.999 [0.981-1.018] |
| 8 | 1.006 [0.989-1.022] | 0.998 [0.977-1.020] | 1.012 [0.993-1.031] |
| 9 | 1.006 [0.989-1.022] | 1.002 [0.981-1.024] | 1.007 [0.988-1.026] |
| 10 | 1.008 [0.991-1.025] | 1.009 [0.987-1.031] | 1.011 [0.992-1.030] |
| 11 | 1.012 [0.996-1.029] | 1.003 [0.981-1.025] | 1.009 [0.990-1.028] |
| 12 | 1.007 [0.991-1.024] | 0.996 [0.974-1.018] | 1.001 [0.982-1.020] |
| 13 | 1.015 [0.999-1.032] | 1.003 [0.981-1.025] | 1.012 [0.994-1.031] |
| 14 | 1.026 [1.010-1.043] | 1.034 [1.011-1.057] | 1.027 [1.008-1.046] |
| 15 | 0.998 [0.982-1.015] | 0.983 [0.963-1.004] | 0.985 [0.966-1.004] |
| 16 | 1.000 [0.984-1.017] | 0.998 [0.978-1.020] | 1.001 [0.982-1.021] |
| 17 | 1.000 [0.984-1.017] | 1.007 [0.986-1.029] | 1.005 [0.986-1.025] |
| 18 | 0.991 [0.974-1.008] | 0.984 [0.963-1.005] | 0.990 [0.970-1.009] |
| 19 | 1.009 [0.992-1.026] | 1.014 [0.993-1.036] | 1.016 [0.996-1.036] |
| 20 | 1.003 [0.986-1.020] | 1.004 [0.983-1.026] | 1.004 [0.984-1.024] |
| 21 | 0.990 [0.974-1.007] | 0.999 [0.979-1.020] | 0.999 [0.979-1.020] |

**Note: The results correspond to Figure 4.**

**Supplementary Material 8. Results for relative risks for all-cause mortality and PM_10_ exposure across a 21-day lagging time window for Valencia based on subgroups Age Group 1 (< 65) and Age Group 2 (≥ 65)**

| Age Group 1 (< 65) | | | |
| --- | --- | --- | --- |
| Lag | Scenario I | Scenario II | Scenario III |
| 0 | 1.004 [0.964-1.046] | 0.994 [0.946-1.044] | 1.004 [0.964-1.046] |
| 1 | 1.014 [0.974-1.056] | 1.031 [0.980-1.085] | 1.014 [0.970-1.061] |
| 2 | 0.962 [0.923-1.002] | 0.936 [0.890-0.986] | 0.948 [0.906-0.992] |
| 3 | 1.024 [0.982-1.068] | 1.049 [0.997-1.103] | 1.045 [0.999-1.092] |
| 4 | 1.010 [0.970-1.052] | 1.012 [0.962-1.065] | 1.002 [0.959-1.048] |
| 5 | 0.983 [0.944-1.024] | 1.010 [0.960-1.063] | 0.975 [0.932-1.019] |
| 6 | 0.950 [0.912-0.990] | 0.954 [0.905-1.005] | 0.948 [0.906-0.992] |
| 7 | 0.964 [0.925-1.004] | 0.980 [0.928-1.034] | 0.977 [0.933-1.023] |
| 8 | 0.964 [0.926-1.003] | 0.976 [0.927-1.029] | 0.982 [0.939-1.028] |
| 9 | 1.004 [0.965-1.044] | 1.008 [0.958-1.061] | 1.013 [0.969-1.060] |
| 10 | 0.994 [0.956-1.034] | 0.994 [0.944-1.048] | 1.009 [0.964-1.056] |
| 11 | 1.004 [0.965-1.044] | 0.997 [0.946-1.050] | 1.018 [0.973-1.066] |
| 12 | 1.021 [0.982-1.062] | 1.029 [0.976-1.084] | 1.028 [0.983-1.075] |
| 13 | 1.022 [0.983-1.062] | 0.998 [0.947-1.052] | 1.004 [0.960-1.050] |
| 14 | 1.034 [0.994-1.075] | 1.004 [0.952-1.059] | 1.023 [0.977-1.070] |
| 15 | 1.027 [0.987-1.067] | 1.012 [0.963-1.064] | 1.014 [0.969-1.062] |
| 16 | 1.016 [0.976-1.057] | 1.005 [0.956-1.057] | 1.000 [0.955-1.047] |
| 17 | 1.001 [0.962-1.042] | 1.000 [0.950-1.051] | 0.992 [0.947-1.039] |
| 18 | 0.983 [0.944-1.024] | 0.974 [0.925-1.026] | 0.981 [0.935-1.029] |
| 19 | 1.000 [0.961-1.042] | 1.013 [0.961-1.067] | 1.014 [0.967-1.064] |
| 20 | 1.009 [0.970-1.050] | 1.013 [0.963-1.067] | 1.004 [0.957-1.053] |
| 21 | 0.986 [0.947-1.027] | 0.975 [0.928-1.025] | 0.975 [0.928-1.025] |

| Age Group 2 (≥ 65) | | | |
| --- | --- | --- | --- |
| Lag | Scenario I | Scenario II | Scenario III |
| 0 | 1.024 [1.006-1.042] | 1.026 [1.004-1.049] | 1.024 [1.006-1.042] |
| 1 | 1.011 [0.993-1.030] | 0.991 [0.969-1.014] | 1.002 [0.983-1.022] |
| 2 | 1.019 [1.001-1.038] | 1.022 [0.999-1.045] | 1.017 [0.997-1.037] |
| 3 | 1.009 [0.991-1.028] | 1.006 [0.984-1.030] | 1.001 [0.981-1.021] |
| 4 | 0.995 [0.977-1.014] | 0.985 [0.963-1.008] | 0.989 [0.969-1.009] |
| 5 | 0.998 [0.980-1.016] | 0.999 [0.976-1.022] | 0.999 [0.979-1.019] |
| 6 | 0.999 [0.981-1.017] | 0.999 [0.976-1.022] | 1.000 [0.980-1.020] |
| 7 | 0.999 [0.981-1.017] | 1.006 [0.982-1.030] | 1.004 [0.984-1.024] |
| 8 | 1.014 [0.996-1.032] | 1.003 [0.980-1.026] | 1.018 [0.997-1.038] |
| 9 | 1.006 [0.988-1.024] | 1.001 [0.979-1.025] | 1.006 [0.986-1.026] |
| 10 | 1.010 [0.993-1.028] | 1.011 [0.988-1.035] | 1.011 [0.990-1.031] |
| 11 | 1.013 [0.996-1.031] | 1.004 [0.981-1.028] | 1.007 [0.987-1.028] |
| 12 | 1.004 [0.986-1.022] | 0.990 [0.967-1.014] | 0.996 [0.975-1.016] |
| 13 | 1.014 [0.996-1.032] | 1.003 [0.980-1.027] | 1.014 [0.993-1.034] |
| 14 | 1.025 [1.007-1.043] | 1.039 [1.015-1.064] | 1.027 [1.007-1.049] |
| 15 | 0.993 [0.975-1.011] | 0.978 [0.956-1.000] | 0.980 [0.959-1.000] |
| 16 | 0.997 [0.980-1.015] | 0.997 [0.975-1.020] | 1.001 [0.981-1.022] |
| 17 | 1.000 [0.982-1.018] | 1.009 [0.986-1.032] | 1.008 [0.987-1.029] |
| 18 | 0.992 [0.975-1.011] | 0.986 [0.963-1.009] | 0.991 [0.971-1.013] |
| 19 | 1.011 [0.993-1.029] | 1.015 [0.992-1.039] | 1.016 [0.995-1.038] |
| 20 | 1.001 [0.983-1.020] | 1.002 [0.979-1.026] | 1.004 [0.983-1.026] |
| 21 | 0.991 [0.973-1.010] | 1.004 [0.982-1.027] | 1.004 [0.982-1.027] |

**Note: The results correspond to Figure 5.**
